# A porcine macrophage cell line that supports high levels of replication of OURT88/3, an attenuated strain of African swine fever virus

**DOI:** 10.1080/22221751.2020.1772675

**Published:** 2020-06-09

**Authors:** Raquel Portugal, Lynnette C. Goatley, Robert Husmann, Federico A. Zuckermann, Linda K. Dixon

**Affiliations:** a The Pirbright Institute, Surrey, UK; b Department of Pathobiology, University of Illinois at Urbana-Champaign, Urbana, IL, USA; c Aptimmune Biologics, Inc., St Louis, MO, USA

**Keywords:** African swine fever virus, macrophage cell line, ZMAC, vaccine, virus replication

## Abstract

The main target cells for African swine fever virus (ASFV) replication in pigs are of monocyte macrophage lineage and express markers typical of the intermediate to late stages of differentiation. The lack of a porcine cell line, which accurately represents these target cells, limits research on virus host interactions and the development of live-attenuated vaccine strains. We show here that the continuously growing, growth factor dependent ZMAC-4 porcine macrophage cell line is susceptible to infection with eight different field isolates of ASFV. Replication in ZMAC-4 cells occurred with similar kinetics and to similar high titres as in primary porcine bone marrow cells. In addition we showed that twelve passages of an attenuated strain of ASFV, OURT88/3, in ZMAC-4 cells did not reduce the ability of this virus to induce protection against challenge with virulent virus. Thus, the ZMAC-4 cells provide an alternative to primary cells for ASFV replication.

## Introduction

African swine fever virus (ASFV) causes a haemorrhagic fever in domestic pigs and wild boar that can result in death of almost all infected animals. The disease is caused by a large DNA virus that is the only member of the *Asfarviridae* family and has a genome length of 170–193 kbp, varying between isolates. ASFV is present in a wildlife cycle in East Africa involving warthogs and soft tick vectors of *Ornithodoros* spp., which are persistently infected with few if any clinical signs. Following the introduction of ASFV in 2007 to Georgia in the Trans Caucasus region, the disease has spread to Russia and further west in Europe infecting a further 11 countries (OIE WAHIS https://www.oie.int/wahis_2/public/wahid.php/Diseaseinformation/diseasehome). In 2018, the first ASFV outbreak was detected in China and rapidly spread over large distances reaching all provinces by the first part of 2019. Further spread to additional countries in Asia has resulted in increased global risk and extended the economic impact of the outbreaks. The absence of a vaccine limits options for control of ASFV. Although efforts to develop a vaccine are increasing, a lack of knowledge about the virus and its interaction with the host hinders this process.

ASFV replicates primarily in cells of the monocyte macrophage lineage which express markers typical of the intermediate to late stages of differentiation [1]. By manipulation of the function of these cells the virus can interfere with and modulate the host’s response to infection. Better understanding of this interaction will provide information on mechanisms of virus immune evasion and pathogenesis, thus underpinning vaccine development. The availability of a biologically relevant cell line to pursue these studies would boost research, by providing a genetically homogenous cell line. The Zuckermann macrophage-4 (ZMAC-4) pig macrophage cell line was derived from foetal pig lung macrophages. This cell line has previously been shown to support replication of Porcine Reproductive and Respiratory Syndrome Virus (PRRSV) to high levels and it was demonstrated that efficacy of vaccine strains produced in this cell line was maintained at a high level following passage [2,3]. The ZMAC-4 cell line was used to investigate modulation of host stress responses to PRRSV infection [4]. In this report we describe experiments which demonstrate that ASFV isolates replicate in the ZMAC-4 cell line to levels similar to primary porcine macrophages without an adaptation step to the cell line. In addition, we show that passage of the natural attenuated ASFV field isolate [5], OURT88/3 in ZMAC-4 cells did not reduce the efficacy of this virus in inducing 100% protection in pigs against challenge with a virulent virus OURT88/1. This isolate had only been grown in primary macrophages previously. These findings indicate the ZMAC-4 cell line provides a suitable cell line for research on ASFV host interactions both in cells and *in vivo* in pigs.

## Materials and methods

### Viruses and cells

The OURT88/3 and NH/P68 genotype I non-haemadsorbing attenuated ASFV strains, virulent strains genotype I OURT88/1, Benin97/1, Georgia 2007/1 genotype II, Malawi LIL 20/1 genotype VIII and moderately virulent strain Dominican Republic [6] strains have been described previously. Other ASFV isolates used were available in the reference collection at Pirbright [5–9]. These viruses were obtained from outbreaks in domestic pigs or isolated from ticks in the field and grown in primary porcine bone marrow cell cultures for a maximum of 3 passages. The OURT88/3 isolate was obtained from domestic pigs in a farm in Portugal. It is a naturally attenuated field isolate that has not previously been grown in cells other than primary porcine macrophages [5].

The cell line ZMAC-4 was derived by lung lavage, from the lungs of a porcine foetus [3] and consists of non-transformed phagocytic cells that require the presence of MCSF to grow. The cell is oligoclonal and stable, as demonstrated by its ability to be successfully passaged for more than 75 times over a period of 8 months without exhibiting a decrease in proliferation capacity. ZMAC-4 cells were from Aptimmune Biologics, Inc., or from the University of Illinois.

### Derivation of the ZMAC-4 cell line

Pig foetuses were harvested on 13 November 2007, from a pregnant sow provided by the secondary specific pathogen-free (SPF) swine herd at the University of Illinois at Urbana-Champaign. The animal use protocol to harvest the porcine foetuses was approved by the University of Illinois IACUC. Specifically, a Genetiporc maternal line (Yorkshire/Landrace) sow (no. 5850), which had been artificially inseminated 60 days earlier with semen from a Duroc boar, was euthanized and its uterus immediately removed using aseptic technique. Without delay, the gravid uterus was transported to a cell culture laboratory, and six foetuses were promptly harvested under sterile conditions. The six foetuses were placed singly on Petri dishes numbered from 1 to 6, and their intact thoracic organs removed. The heart, oesophagus and other membranes were dissected away from the respiratory organs ensuring that the lung and trachea remained connected and intact. The surface of the lung was thoroughly rinsed with sterile Hank’s balanced salt solution (HBSS) to remove any visible blood, and transferred to an empty Petri dish. Cells within the airways were harvested by bronchoalveolar lavage (BAL) by gently instilling into the lung 10 mL of sterile HBSS via the trachea with the aid of an 18G × 1 in. needle attached to a 10 mL luer-lock tip syringe. Viable mononuclear cells obtained from the resulting BAL were isolated by isopycnic centrifugation as previously described [10]. The few hundred cells recovered from each foetal lung were suspended in 3 mL of complete culture medium, consisting of RPMI-1640 media supplemented with 10% foetal bovine serum, sodium pyruvate (1 mM), and non-essential amino acids (1×). The entire 3 mL cell suspension from each foetus was separately seeded into a single well of a 6-well plate for suspension culture (Sarstedt) and cultured at 37°C in an atmosphere of 5% carbon dioxide in air saturated with water vapour. Eight days later, the cultures were fed with 3 mL of fresh culture medium. At 16 and 24 days after initiating the culture, the cell population obtained from foetus number 4 exhibited a sufficient increase in cell number to warrant transferring half the volume of the culture into an additional well of a 6-well plate and feeding the original and the new well with an equal volume of fresh culture medium. This process was repeated as needed. The growth of the ZMAC-4 cell line was evident by the formation of colonies growing in suspension, as well as loosely attached to the surface of the culture vessel. The cell line derived from foetus number 4 was termed Zuckermann macrophage (ZMAC)-4. Beginning at 37 days after initiation of the culture, the expansion of the ZMAC-4 cell line was aided by the supplementation of the medium with 10 ng/ml of recombinant murine macrophage colony stimulating factor (M-MCSF; Sigma-Aldrich Product No. M9170). Further expansion of the ZMAC-4 cell line was accomplished by transferring cells into T75 tissue culture flasks for suspension culture (Sarstedt). Beginning at approximately 4 months after its isolation, the ZMAC-4 cell line had expanded sufficiently to enable freezing of several batches of cells over a period of 5 months. Each batch consisted of 10–20 vials with at least 5 × 10^6^ cells per vial. A master cell stock (MCS) was created from the batch of ZMAC-4 cells frozen on 26 March 2008, which has been approved by the USDA Center for Veterinary Biologics for commercial vaccine production. The ZMAC-4 cells used for this study were derived from a working cell stock of the USDA approved ZMAC-4 MCS. The approximate population doubling level (PDL) of the ZMAC-4 cell line was calculated as described by [11]. All of the ZMAC-4 cell cultures used for virus culture in this study were performed with cells at an approximate population doubling level (PDL) since isolation ranging from 65 to 69.

### ZMAC-4 cell line method of culture and cell doubling time

Starting with a frozen vial of ZMAC-4 cells containing 5 × 10^6^ cells, and after quick thawing, the contents of the vial are transferred to a 15 mL conical centrifuge tube containing 12 mL of culture medium. Following centrifugation at 1500 RPM for 10 min, the resulting cell pellet is resuspended in 5 mL of culture medium and transferred to a T75 tissue culture flask (Sarstedt) containing 45 mL of complete culture medium supplemented with 10 ng/ml of m-MCSF (Shenandoah Biotechnology, Inc.). Under these culture conditions, the ZMAC-4 cells form colonies that are loosely attached to culture surface, and become readily suspended into the medium by gently shaking the culture vessel. Thus, the culture of ZMAC-4 cells can be described as a “stationary suspension culture.” Hence, the feeding of ZMAC-4 cells entails adding a volume of complete culture medium supplemented with 20 ng/mL of M-MCSF equal to the volume present in the culture vessel being fed. Spilling of culture medium from the flask, as a result of the gradual increase in volume from feeding, is avoided by placing the T75 flask on the incubator shelf at a 20^°^ angle. This is accomplished by resting the end of flask, where the flask’s mouth is located, on a 2-inch-tall tissue culture bottle cap. Once the volume of the culture exceeds the capacity of the culture vessel to hold the culture fluid (about 150 mL for a T75), a new T75 flask can be established by transferring half the volume of the existing flask to the new flask and feeding each flask with 75 mL of complete medium supplemented with 20 ng/mL of M-MCSF. Under optimal conditions, a cell suspension of 0.6–0.8 × 10^5^ cells/mL, obtained after feeding an existing cell suspension with an equal volume of fresh medium, would be expected to reach a concentration of 1–1.3 × 10^5^ cells/mL three days later. The doubling time of the ZMAC-4 cell line was determined using the formula described by [11] with data collected from five independent cell expansion runs using cells in an exponential phase of growth. Counting of the ZMAC-4 cells was performed with a Moxi Z Automated Cell Counter (Orflo Technologies) using Type S cassettes.

### Flow cytometry

Staining of suspensions of ZMAC-4 cells for flow cytometry consisted of incubations in Flow PBS (PBS, 1.0% BSA, 0.01% sodium azide) containing the indicated mAbs for 30 min at ice-cold temperature with each step being terminated by one wash with Flow PBS. Initially, ZMAC-4 cells were left untreated or were separately exposed to one of the following monoclonal antibodies that recognize the indicated porcine molecule [12,13]: CD14 (biG 10/14), CD163 (2A10/11), CD172 (74-12-55), CD203a (PM18-7), PU.1 (E.388.3). Afterwards, the cells were sequentially incubated in Flow PBS containing goat anti-mouse Ig conjugated to PE (Southern Biotech), 2% normal mouse serum (Sigma) and 100 mg/ml mouse IgG (Zymed Laboratories, Invitrogen). To detect PU.1, cells were fixed with paraformaldehyde and permeabilized with detergent as previously described [14]. To detect phagocytic activity, ZMAC-4 cells were incubated in the presence of Fluoresbrite YG microspheres 2.00 micrometer (Polysciences, Inc) for 30 min at 37°C in culture medium. As a control, ZMAC-4 cells were incubated with the same particles at ice cold temperature in the presence of 1% Sodium Azide. Background fluorescence in this assay was detected using cells not exposed to the microspheres. Flow cytometric analysis was performed with an LSR II flow cytometer (BD Biosciences, San Jose, CA, USA). Data analyses and preparation of graphical representations were done with FlowJo software (Ashland, OR, USA).

### Virus infection and titration

ASFV was titrated by limiting dilution in cultures of porcine bone marrow cells and infection detected by haemadsorbtion assay (HAD) or by immunofluorescence using a monoclonal antibody against the p30/pCP204L protein as described previously [15]. Evaluation of ASFV growth in ZMAC-4 cells was carried using a multiplicity of infection (MOI) 0.05 of Georgia 2007/1 and the cells were frozen at −80°C after the different infection times. The cells were subjected to two freeze–thaw cycles before titration in parallel on porcine bone marrow cells from the same pig. Titres, as TCID_50_/ml, were obtained by immunofluorescence as stated above and HAD_50_/ml were obtained by addition of erythrocytes, collected from heparinized pig blood, together with the inoculum. Titrations (in triplicate wells) were made in parallel using cells from the same culture passage.

### Virus genome detection

DNA was extracted from whole blood collected in EDTA or tissues collected at post-mortem and detected by qPCR as described previously [16,17].

### Animal experiments

Experiments were carried out in Specified Animal Pathogens Order 2008 UK (SAPO4) high containment facilities at The Pirbright Institute and regulated by the Animal (Scientific Procedures) Act UK 1986. Large White and Landrace cross-bred pigs 8–9 weeks old (18–22 kg) from a high health status farm were used in the experiment.

## Results

### Features of ZMAC-4 cells

**Figure 1.**
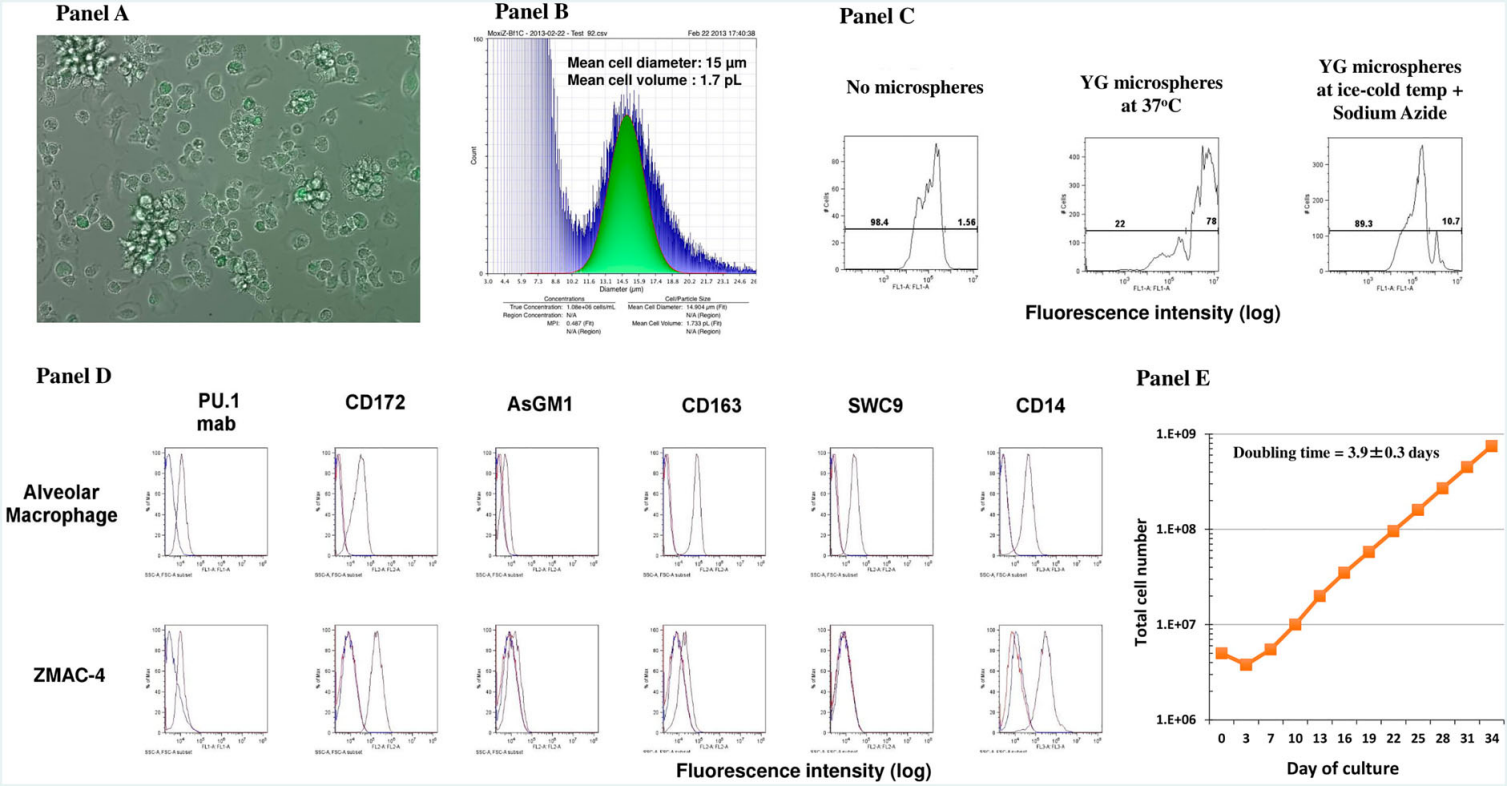
Features of ZMAC-4 cells. (A) Morphology of ZMAC-4 cells. Live cultures of ZMAC-4 cells were imaged with an inverted phase contrast microscope. Original magnification is 40×. Note the presence of filipodia and lamellipodia. (B) Mean cell diameter and volume of ZMAC-4 cells as determined with a Moxi Z Automated Cell Counter using a Type S cassette. The graph represents the analysis of 75,000 cells. (C) Phagocytic activity of ZMAC-4 cells. Flow cytometric analysis of cells that were left untreated (no beads), or exposed YG microspheres for 30 min at either 37°C or at ice cold temperature in the presence of 1% Sodium Azide. (D) Flow cytometric analysis of primary porcine alveolar macrophages (top row) or ZMAC-4 cells (bottom row) reacted with antibodies specific for the indicted molecule. (E) Representative continuous expansion of ZMAC-4 cells starting from frozen cells. The indicated doubling time was calculated using data from five independent experiments with cells that were in exponential growth and using the formula developed by Hayflick [11].

Morphologically the ZMAC-4 cells exhibit the presence of filipodia and lamellipodia (Figure 1A), and have a mean cell diameter of 15 μM (Figure 1B). The ZMAC-4 cells are phagocytic (Figure 1C), and uniformly express several surface markers characteristic of porcine alveolar macrophages (PAM) including CD14, CD163, and CD172, as well as E-twenty-six (E26)-family transcription factor PU.1 (Figure 1D). PU.1 is a transformation-specific family transcription factor that plays a pivotal role in normal myeloid differentiation [18]. It is most prominent in myeloid cells and involved in the development and maturation of B cells, macrophages, and neutrophils. Thus, the expression of PU.1 is a hallmark of macrophages. Notably the ZMAC-4 cells do not appear to express SWC9/CD203a. The lack of CD203a likely indicates that the ZMAC-4 cells are not as mature as primary alveolar macrophages. This is expected since the ZMAC-4 cells are able to proliferate, while primary alveolar macrophages do not. As described in the materials and methods section, the culture system that works best for culturing the ZMAC-4 cell line is a stationary suspension culture, using RPMI-1640 culture medium supplemented with FBS, sodium pyruvate, non-essential amino acids and MCSF. A representative expansion of ZMAC-4 cells, starting from five million frozen cells that were expanded continuously for 34 days, is shown in Figure 1E. In this culture system, the volume of the cell culture was gradually increased every three days by simply adding complete culture medium to the tissue culture vessel equal to the volume present in the flask at the time of feeding. The culture was gradually expanded to additional flasks as needed. After 34 days of continuous culture, a >100-fold expansion from the initial cell number was achieved. Using data from five independent expansion runs the doubling time for the ZMAC-4 cell line was estimated to be 3.9 ± 0.3 days (Figure 1E). Previous results [4] showed that the ZMAC-4 cells produce IFN-α in response to polyIC with similar kinetics as PAMs. Thus, the ZMAC-4 cells exhibit a number of characteristics typical of PAMs and were predicted to be susceptible to ASFV infection.

### ASFV replicates to similar titres in ZMAC-4 cells as primary porcine bone marrow cells

In order to test the susceptibility of ZMAC-4 cells for infection by field isolates of ASFV, infections in these cells were compared with primary macrophage cultures derived from pig bone marrow (PBM). To assess the infection susceptibility with field isolates of ASFV, the same virus stocks of 8 field isolates of ASFV (OURT88/3, NH/P68, Benin 1997/1, Georgia 2007/1, Malawi LIL20/1, Tengani, MOZ 94/1, ZOM 2/84, Dominican Republic) from genotypes I, II, VIII were titrated in triplicate in parallel in ZMAC-4 and PBM cells. These isolates were from Pirbright Institute Reference Collection and had not previously been grown in cells other than primary porcine macrophages [8]. The results in Table 1 show that titrations in ZMAC-4 cells reached similar levels in comparison to those in PBM cells for all the isolates tested. These results indicated that the ZMAC-4 cells are similarly susceptible to infection as primary porcine macrophages.

**Table 1.** Titration of different ASFV isolates on primary porcine bone marrow macrophages and ZMAC cells.

	Genotype	PBM (TCID50/ml)	ZMAC (TCID50/ml)
OURT 88/3	I	3.16 × 10^7^	6.81 × 10^7^
NH/P68	I	3.16 × 10^3^	3.16 × 10^5^
Benin 1997/1	I	1.47 × 10^7^	3.16 × 10^7^
Georgia 2007/1	II	3 × 10^6^	3 × 10^6^
Malawi LIL 20/1	VIII	7 × 10^6^	7 × 10^6^
Tengani		3 × 10^6^	1 × 10^7^
MOZ 94/1	II	1 × 10^7^	1 × 10^7^
ZOM 2/84	VIII	3 × 10^5^	3 × 10^4^
Dominican Republic	I	3 × 10^5^	3 × 10^4^

Data in the table describes the genotype of each of the isolates titrated in parallel on adherent PBM cultures and ZMAC cells.

**Figure 2.**
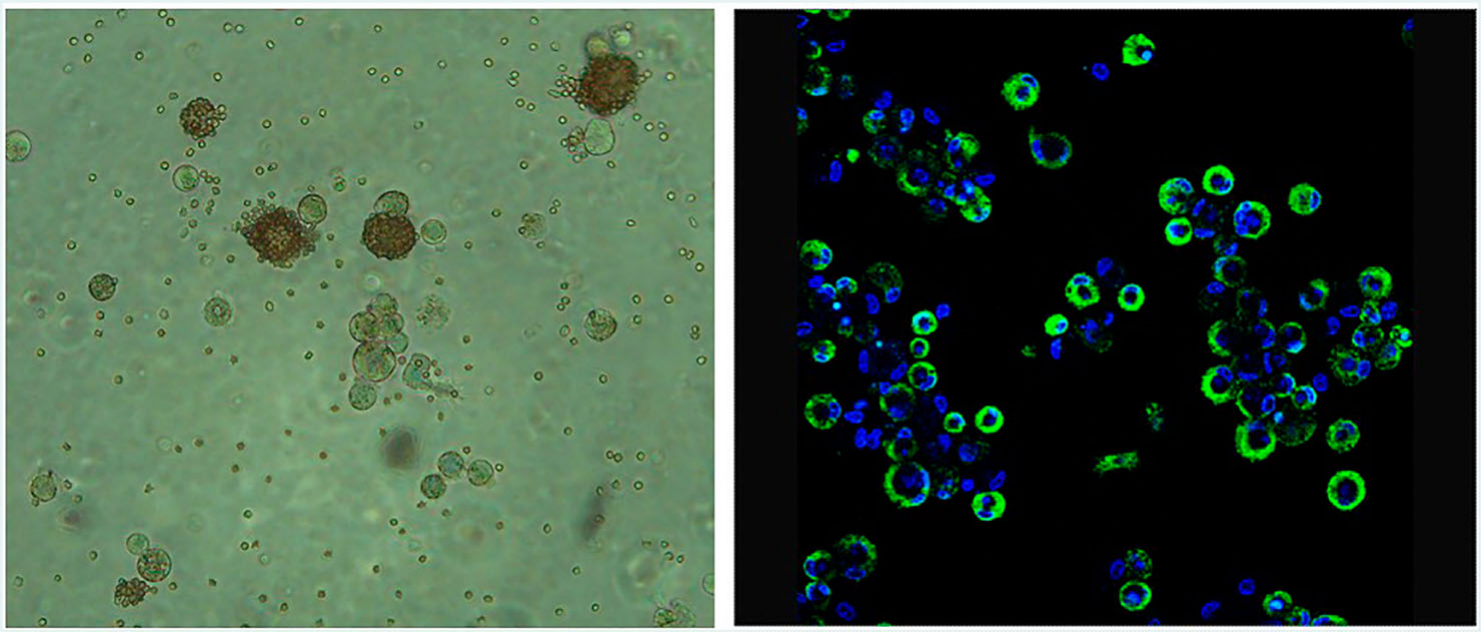
Infection of ZMAC-4 cells with ASFV Panel A. Haemadsorption of ZMAC cells infected with ASFV Benin 1997/1. Erythrocytes were added to the cells together with the viral inoculum. Presence of haemadsorption rosettes were screened after three days of culture. Panel B. ZMAC cells were infected at high multiplicity with the Malawi LIL20/1 isolate of ASFV and at 18 h post-infection fixed. Fixed cells were permeabilized with non-ionic detergent and stained with a monoclonal antibody against the early virus protein p30 (in green) followed by Alexa Fluor conjugated anti-mouse secondary antibody. DNA was stained with DAPI in blue. Cells were visualized by confocal microscopy. Greater than 80% of cells show green staining indicating they are infected.

To determine the effectiveness of titration by haemadsorption (HAD/ml) in ZMAC-4 cells compared to indirect immunofluorescence (against viral early protein P30 – TCID50/ml), different field isolates of ASFV NH/P68, Georgia 2007/1 and Benin 97/1 were titrated in parallel on ZMAC-4 cells with erythrocytes added to the culture medium. The virus stocks had not previously been grown in cells other than primary porcine macrophages. The cells were observed under the microscope 3 days after inoculation similarly to previous titrations. Presence of haemadsorbing cells at different inoculum dilutions was screened (Figure 2 and unpublished results) and the titres obtained (Table 2) showed very similar results using both titration techniques except for the non-haemadsorbing isolate NH/P68, which as expected didn’t induce haemadsorption. This isolate has interruptions in the genes coding for the CD2v/EP402R and C-type lectin/EP153R genes explaining its non-haemadsorbing phenotype [6]. The images shown in Figure 2 were obtained as examples to illustrate detection of infection by HAD or IF and using different cultures and inoculation titres. The cultures used for titrations of different virus isolates shown in Table 2 were different from those shown in Figure 2. The results shown in Table 2 were obtained from cultures with the same cell numbers and same batch of either ZMAC-4 or PBM cells.

**Table 2.** Titration of ASFV field isolates on ZMAC-4 cells by indirect immunofluorescence (TCID50) and haemadsorption (HAD50).

	TCID50/ml	HAD/ml
NH/P68	3.16 × 10^5^	0
Georgia 2007/1	3.16 × 10^6^	7.9 × 10^6^
Benin 1988/1	3.16 × 10^7^	1.83 × 10^7^

The different ASFV isolates were titrated in parallel on PBM cultures or ZMAC-4 cells. For TCID50 titres the cells were stained for immunofluorescence detection of P30 early viral protein and observed under the fluorescence microscope; for determining titres as HAD50 inoculum was added to the cells together with pig erythrocytes and screened under the optical microscope for the presence of haemadsorption rosettes at the different dilutions.

### Dynamics of ASFV replication in ZMAC-4 cells compared to PBM cells

**Figure 3.**
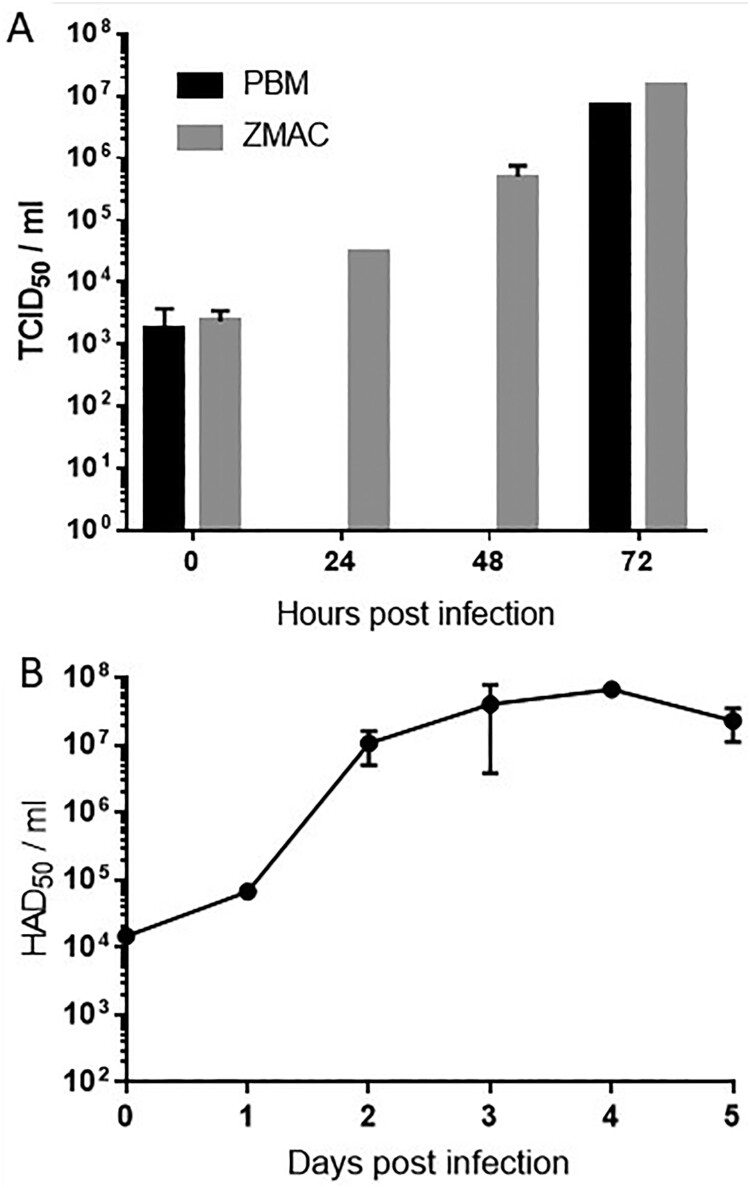
Growth of ASFV in ZMAC-4 cells and primary porcine macrophages. Panel A: ASFV growth in ZMAC cells and in primary macrophage cultures. Georgia 2007/1 was used to infect similar numbers of ZMAC cells and PBM adherent cells at MOI 0.05 in duplicate. At the different time points after infection aliquots of the cultures (cells plus supernatant) were titrated on same origin PBM cultures in parallel (titres determined as TCID50/ml). Panel B: ASFV growth in ZMAC cells over 5 days. Georgia 2007/1 was used to infect ZMAC cells at MOI 0.05. At different time points after infection up to five days aliquots of the cultures (cells plus supernatant) were titrated on same origin PBM cultures in parallel (titres determined as HAD50/ml).

Virus progeny production was compared after infections on PBMs and ZMAC-4 cultures. Infections were made with the same virus stock of Georgia 2007/1 at low MOI (0.05) in both cell types and virus growth was monitored at 0, 24, 48 and 72 h in the ZMAC-4 cells and at 0 and 72 h in PBMs (Figure 3A). ZMAC cells or PBMs (2 × 10^5^) were inoculated at a multiplicity of infection of 0.05 with Georgia 2007/1 ASFV isolate in a volume of 0.5 ml medium. The inoculum was present throughout culture. After the times indicated virus was recovered from the cells and supernatant and titres present determined by haemadsorbtion assay in PBMs. Titres in ZMAC-4 cells increased progressively and reached approximately the same titres as the PBM cultures after 72 h (1.47 × 10^7^TCID_50_/ml in ZMAC-4 and 6.81 × 10^6^ TCID_50_/ml in PBM), confirming the high susceptibility of the ZMAC-4 cells to ASFV infection and effective production of the virus. The Georgia 2007/1 isolate had not previously been grown in cells other than primary porcine macrophages confirming results described in Table 2 that an adaptation to ZMAC-4 cells was not required. To assess how long the ZMAC-4 cultures could sustain ASFV growth we infected cells at a low MOI 0.05 and measured virus progeny production over a period of five days (Figure 3B). Progeny virus titres increased more than 3 Log units by day 2 after inoculation and these values were maintained until day 4 of culture (from 1.48 × 10^4^ to 6.76 × 10^7^ HAD_50_/ml), decreasing slightly at day five (2.32 × 10^7 ^HAD_50_/ml).

### Passage of ASFV live-attenuated strain OURT88/3 in ZMAC-4 cells does not reduce the induction of a protective response against challenge with virulent ASFV in pigs

To determine if passage of ASFV in ZMAC-4 cells altered immunogenicity of the virus in pigs, we cultured the attenuated genotype I strain OURT88/3 for 12 passages in ZMAC-4 cells using a low multiplicity of infection (MOI of 0.1). This was carried out in ZMAC-4 cells cultured in 6 well plates. The cells were freshly split before infection. Titres of virus obtained were approximately 10^6^ per ml after 3 days culture in each passage. This was similar to titres obtained in primary macrophage cultures. Thus, there was no evidence that an adaption of the virus was required to replicate in ZMAC-4 cells. The virus harvested after the 12th passage was used to immunize pigs as described below.

**Figure 4.**
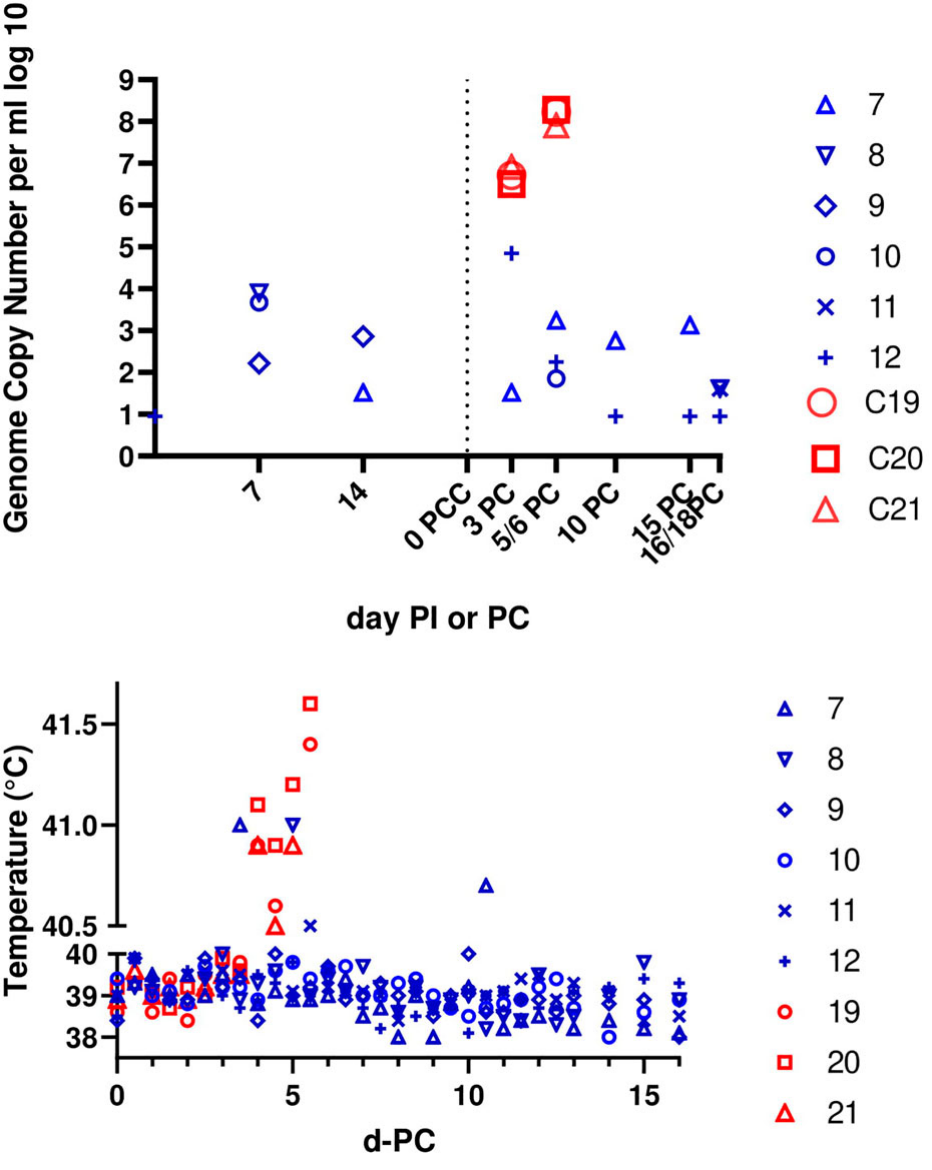
Immunization of pigs with OURT88/3 passaged 12 times in ZMAC-4 cells. (A) Shows on the *y* axis a log 10 scale (in blue numbers 7–12) genome copies per ml of whole blood in pigs immunized with ZMAC cell-passaged OURT88/3 before and after challenge with virulent OURT88/1 strain. The levels in control non-immune pigs are shown in red (C19, C20, C21). The *x*-axis shows days post-immunization (PI) or post-challenge (PC). (B) Shows the rectal temperature on the *y*-axis of different immunized pigs (blue 7–12) post-challenge and of the control non-immune pigs (red 19, 20, 21).

A group of 6 pigs was inoculated intra-muscularly with 10^4^ TCID_50_ of the OURT88/3 isolate that had been passaged 12 times in ZMAC-4 cells (Pigs 7-12). In parallel a group of 6 pigs was inoculated with 10^4^ TCID_50_ of the same stock of OURT88/3 which had not been passaged in ZMAC-4 cell (Pigs 13-18). Results from this group of pigs have previously been reported [17] and the comparison with the group immunized with ZMAC-4 passaged virus is shown on Table 3. Pigs were monitored daily for clinical signs and these were scored as described previously [16]. At 21 days post-inoculation the pigs were challenged with virulent isolate OURT88/1 intramuscularly with 10^4^ TCID_50_ in parallel with a control group of 3 non-vaccinated pigs (Figure 4).

**Table 3.** Clinical signs and viremia before and after challenge in pigs immunized with OURT88/3 passaged in ZMAC-4 cells or the parental stock before passage.

	Clinical signs		ASFV genome copies/ml in blood				
	Pre-challenge	Post-challenge	Pre-challenge	Level	Post-challenge	Level	Protection
OURT88/3	3/6 transient swollen joints	None	4/6	10^2–3.5^	4/6	10^2–4^	6/6
OURT88/3 ZMAC-4	2/6 transient skin or joint swelling	1 pig 40.5 temperature 1 day	4/6	10^2–4^	4/6	10^2–5^	6/6

Clinical signs and virus genome detected in blood before and after challenge in groups of 6 pigs immunized with ZMAC-4 cell-passaged OURT88/3 described here and in a parallel previously described experiment [14] using the same virus stock before passage.

All of the pigs immunized with the OURT88/3 passaged in ZMAC-4 cells survived the challenge and were euthanized between 16 and 18 days post-challenge. Clinical signs were very low post-immunization (Figure 4(B)). Pig 11 had a temperature of 40.0°C on day 3 post-immunization and 40.2°C on day 15 post-immunization. No other temperatures above 40.0°C were observed. After challenge pig 7 developed a temperature of 41°C at day 3 post-challenge and 40.7°C at day 10 post-challenge. Pig 9 had a temperature of 40°C at day 5 post-challenge. Pig 10 had a temperature of 40.1°C on the day of challenge, and pig 11 had 40.1°C on day 4 and 40.5°C on day 5 post-challenge. All pigs survived until termination at day 16–18 post-challenge (Figure 4B). Two pigs (9 and 11) developed skin swellings or lameness and were treated with antibiotics which resolved these issues. Post-mortem examination showed no macroscopic lesions typical of ASF in this group of pigs.

In contrast, the control non-vaccinated pigs all developed temperatures above 40.5°C by day 4 post-challenge, accompanied by lethargy and partial or complete loss of appetite. In addition all showed skin reddening around the ear. All pigs in this group (pigs 19, 20, 21) were euthanized on day 5 post-challenge. Post-mortem examination revealed signs typical of acute ASFV including enlarged and haemorrhagic spleens and haemorrhages in several lymph nodes.

Blood samples were collected at weekly intervals before challenge and at 3 day intervals post-challenge to measure levels of virus genome by qPCR (Figure 4A). The results showed four pigs (7, 8, 9, 10) from the group immunized with the OURT88/3 passaged in ZMAC-4 cells had low levels of virus genome (between 10^2^ and 10^4^ genome copies/ml) in blood before challenge. After challenge 4 pigs had detectable virus genome. Of these pigs number 12 had 10^5^ genome copies/ml at day 3 post-challenge and all other positive samples were 10^3^ genome copies/ml or lower. Analysis of samples from spleen and tonsil detected no virus genome in tissues from any of the immunized and challenged pigs. In contrast pigs in the control group developed high levels of infectious virus in blood at day 3 post-challenge (3 to 8 × 10^6 ^TCID_50_/ml) rising to 8 × 10^7^ to 2 × 10^8^ TCID_50/_ml by day 5 or 6 post-challenge. The control pigs also had high levels of virus genome in spleen at termination (5 × 10^3^ to 2.7 × 10^4^ genome copies per mg). In conclusion pigs immunized with the OURT88/3 passaged in ZMAC-4 cells were all protected against challenge with the virulent isolate OURT88/1.

## Discussion

The lack of porcine macrophage cell lines resembling the *in vivo* target cells for ASFV replication has restricted research on the virus host interactions, limited the development of live-attenuated vaccines and made virus diagnosis more complex. The main target cells for ASFV replication in vivo are monocytes and macrophages. In cell culture those cells that express markers characteristic of intermediate and late stages of differentiation are susceptible to infection [1]. The morphology and expression of cell surface markers on ZMAC-4 cells confirmed their phenotype was similar to primary alveolar macrophages. For example, expression was confirmed of the PU.1 transcription factor, which is considered a hallmark of macrophages.

Here we show that, as predicted from their phenotype, the ZMAC-4 porcine macrophage cells are highly susceptible to infection with ASFV field isolates without an adaptation process and that the virus replicates with similar dynamics and to similar titres as in primary porcine macrophages. During an adaptation process to cell culture it is expected that virus would initially infect a small percentage of cells and replicate slowly but that this may increase during passage. Virus adaptation to established cell lines often results in genomic changes and reduced virus growth in the original primary cells. For example, adaptation of an ASFV field isolate to Vero cells resulted in multiple virus genome changes and loss of ability to replicate in primary porcine macrophages or to induce disease in pigs [19].

ASFV codes for multiple copies of several multigene families and variation in numbers of these genes occurs during the evolution of the virus in its natural hosts [20–22]. Depending on the genes deleted this can result in virus attenuation in pigs. For example, the OURT8/3 isolate has a deletion of several copies of MGF 360 and MGF 505 [6]. Targeted deletion of these genes from a virulent isolate resulted in virus attenuation and increased induction of type I interferon but did not result in reduced replication of the virus in primary macrophages [23]. Thus, the genes deleted from the OURT88/3 genome have been implicated in inhibiting induction of type I interferon but not reduction of the ability of the virus to replicate in macrophages. A further example of the natural evolution of ASFV is provided by analysis of another natural attenuated ASFV field isolate obtained from wild boar in Estonia had a large genome transposition from one genome end to the other and a large genome deletion including multiple members of MGF genes [24].

It is not surprising that loss of members of multigene families can occur as one of the many genetic changes observed during the adaptation of ASFV to established cell lines including Vero cells [6,19]. Since variation in numbers of MGF members has not been associated with loss in ability of ASFV to replicate in macrophages, it is likely that some of the many other genome changes, including additional deletions and mutations, observed are important for this adaptation to cell culture.

Although CD163 was first suggested to be required for infection by ASFV, subsequent studies showed that monocytes that didn’t express CD163 could also be infected. Gene-edited pigs lacking CD163 were fully susceptible to infection by ASFV. Monocytes and macrophages from these pigs were also susceptible to infection confirming that CD163 is not required [25]. The availability of a suitable macrophage cell line will facilitate research by providing a genetically homogenous source of cells avoiding the large variation associated with primary cells from different outbred pigs. Research directed at understanding host factors that restrict virus replication, and virus modulation of host cell function including evasion of host defences will also be facilitated. In addition, studies on virus replication and host responses in pigs can be undertaken.

Importantly availability of a porcine macrophage cell line can also replace the need for primary porcine cells for virus diagnosis and isolation. In our studies we showed that titration of virus in ZMAC-4 cells gave very similar results to primary porcine bone marrow cells and that both the gold standard haemadsorption assay and immunofluorescence assay for measuring virus titres can be used with these cells. Thus, the ZMAC-4 cells can be used as an alternative to primary porcine macrophage cultures for ASFV titration and detection.

We also demonstrated that 12 passages of the live-attenuated ASFV strain OURT88/3 in ZMAC-4 cells did not alter the safety of the virus following immunization of pigs by the intramuscular route as compared to the parental OURT88/3 virus [17]. All immunized pigs were also protected following lethal challenge with highly virulent genotype I strain Benin 97/1. The results were similar to other experiments in which OURT88/3 was delivered by the same route and dose [5,23,26]. We showed that OURT88/3 and several other field strains from different genotypes of ASFV grow to high titres in ZMAC-4 cells. Together the results confirm that the ZMAC-4 cells provide a realistic alternative to primary macrophages or other cell lines for ASFV research and have good potential for use in diagnosis and vaccine production.
